# Machine learning-based classification model to differentiate subtypes of invasive breast cancer using MRI

**DOI:** 10.3389/fonc.2025.1588787

**Published:** 2025-06-03

**Authors:** Nadesalingam Paripooranan, Warnakulasuriya Buddhini Nirasha, H. R. P. Perera, Sahan M. Vijithananda, P. Badra Hewavithana, Lahanda Purage Givanthika Sherminie, Mohan L. Jayatilake

**Affiliations:** ^1^ Department of Radiology, Faculty of Medicine, University of Peradeniya, Peradeniya, Sri Lanka; ^2^ Department of Radiography/Radiotherapy, Faculty of Allied Health Sciences, University of Peradeniya, Peradeniya, Sri Lanka

**Keywords:** machine learning, breast MRI, invasive breast cancer, invasive ductal carcinoma, invasive lobular carcinoma

## Abstract

**Introduction:**

Breast cancer is considered one of the most lethal diseases among women worldwide. Invasive Ductal Carcinoma (IDC) and Invasive Lobular Carcinoma (ILC) are the two most prominent subtypes of breast cancer. They differ in epidemiology, molecular alterations, and clinicopathological features. Patient treatment and management also differ due to these variations.

**Aim:**

The study aimed to develop a predictive model to differentiate IDC and ILC using machine learning techniques based on the morphological features of the contralateral breast. Methods- 143 magnetic resonance imaging (MRI) images were sourced from the “DUKE Breast-Cancer” collection on the Cancer Imaging Archive website. Regions of interest were drawn on each slice to compute the morphological features of the contralateral breast using the 3D Slicer application. Supervised learning methods were applied to the morphological features to build a predictive model incorporating a Random Forest Classifier to differentiate IDC and ILC. Hyperparameters were tuned to optimize the model.

**Results:**

The model was able to differentiate IDC and ILC with an accuracy of 79% and an Area Under the Curve of 0.851 on the Receiver Operating Characteristic Curve. Among the morphological features, the total volume of the contralateral breast, surface area of the contralateral breast, breast density, and the ratio of the total volume of the contralateral breast to its surface area had higher F-scores, indicating that the dimensions of the contralateral breast could be an important factor in differentiating IDC and ILC.

**Conclusion:**

This study successfully developed and optimized a predictive model based on breast morphological features to differentiate IDC and ILC using machine learning methods.

## 1 Introduction

Breast cancer is the most common malignancy among women worldwide and also the second most common cancer overall, following only lung cancer (1). According to the World Health Organization (WHO), approximately 2.3 million women were diagnosed with breast cancer in 2020, and it accounted for an estimated 685,000 deaths globally (2). Despite advancements in early detection, it is projected that in 20 years, more than 60% of new breast cancer cases and 70% of deaths will occur in low and middle income countries (3).

Breast cancer is a heterogeneous disease, primarily originating from the inner lining of the breast’s fibroglandular tissues, which are responsible for milk production and supply. Heterogeneity can be reflected in the diversity of histological and molecular subtypes of breast cancer (4). Invasive breast cancer (IBC) is one of the major histological categories of breast cancer and can be further divided into various subtypes. IBC cells are proficient in invading through the fibroglandular tissue wall into the surrounding connective and fatty tissues. However, it should be noted that IBC can present without necessarily spreading beyond the breast and adjacent lymph nodes (5). The study of the different characteristics of IBC subtypes can lead to understanding which strategies will be more effective in developing therapies that could improve patient outcomes.

Invasive ductal carcinoma (IDC) and invasive lobular carcinoma (ILC) are the two most common types of IBC that differ in their epidemiology, molecular alterations, clinicopathological features, and natural history (6). ILC is the second most common type after IDC. IDC occurs in approximately three-fourths of patients, while ILC occurs in less than one-tenth of patients (7). ILC is differentiated from IDC with increased frequency of multifocality, bilaterality, multicentricity, and difficulty in determining its margins during clinical examinations (8, 9). Past research has shown that ILC patients were older (10). This could be due to the low proliferative rate or difficulty detecting ILC (11). Tumor size was found to be larger in ILC (11, 12). The incidence of contralateral breast cancer was also higher in ILC (11). The metastatic pattern was also found to be different for ILC and IDC since ILC is less likely to affect the lungs, pleura, and CNS compared to IDC (11). Due to these differences, patient management also differs between IDC and ILC. For ILC patients, conservation therapy is not recommended unless it is early-stage due to the multicentric, multifocal characteristic. Radiation therapy controls the microscopic foci of multicentric disease, as mentioned in several studies (13). The neoadjuvant setting was used in several retrospective studies, which have shown that patients with IDC benefit much more from chemotherapy than those with ILC (14, 15).

Breasts are composed of glandular, fibrous, and adipose tissues. The glandular tissues form the lobules and ducts, which are the primary components of milk production. The lobules are present in the background of fibrous, and adipose tissues. Adipose tissue plays an integral role in the morphogenesis of the breast and participates in mammary epithelial cell differentiation (16). Breast volume and breast density, two features of this study, are related to the dimensions of these tissues. Breast density refers to the ratio of fibroglandular tissues to fatty tissues of a patient’s breast and may vary between individuals and over the course of a patient’s life (17). It is considered an independent risk factor for breast cancer (18). Breast volume has been suggested as a controversial risk factor for the last few decades. The study by Li et al. found that breast cancer might not be entirely related to breast volume (19). But, according to the study by Schutt et al., breast size was correlated to the occurrence of breast cancer (20).

The total breast volume is calculated using several methods such as the water displacement method, three-dimensional Ultrasound scan, mammography, Computed Tomography, and Magnetic Resonance Imaging. Among these, MRI is considered the modality with the highest accuracy (16). According to recent studies, breast MRI is considered a superior modality for measuring fibroglandular tissue volume and breast density because of its ability to segment the whole breast from the body and segment fibroglandular tissues within the breast, slice by slice. Breast MRI has become a revolutionary investigative tool in the diagnosis of breast cancer due to its better performance compared to mammography and US (21). Breast MRI adds anatomical characterization, information on tumor vascularity and perfusion dynamics, and excellent lesion detection. Even though breast MRI provides sensitivity above 88.1%, its specificity fluctuates between 83% and 98.4%, critically dependent on observer experience (22). The sensitivity of breast MRI is usually higher than 95%, making it vital for detection before symptoms emerge, especially in cases where other imaging modalities are not precise enough. Similarly, it has higher sensitivity in detecting breast cancers in denser breasts (21).

The main challenges in breast cancer screening and imaging diagnosis are the complexity and wide range of image features, varying quality of images, and inconsistent interpretations by different radiologists and medical institutions. Artificial Intelligence could be incorporated to increase the accuracy and precision of these interpretations. Furthermore, personalized predictions and recommendations derived from individual patient data through machine learning (ML) can optimize treatment strategies, enhancing overall patient care. By handling complex, high-dimensional datasets, ML algorithms can extract relevant features from MRI images, facilitating the creation of robust classification models. Several ML algorithms are popular in this field. The Random Forest Classifier (22–24), Support Vector Machine (25), and k-nearest neighbor (26) are some of the algorithms used in medical image processing and building predictive models. The Random Forest Classifier is a collection of decision trees where each tree is trained individually with sub datasets randomly, and the outputs of all the individual trees are combined to make a prediction (27). In the training phase, a large number of decision trees are created initially, which is called bootstrap aggregating. Each tree is trained on a random subset of the data and grows until it reaches its maximum depth. In the testing phase, each case is passed through all the trees in the forest and a class prediction is given for each tree. The final output is determined by the majority vote. Jacques Wainer conducted a comprehensive study on 14 classification algorithms to determine which algorithm performs best under specific conditions. Random Forest along with, Gradient Boosting Machines, and Radial Basis Function Support Vector Machines were the top performing algorithms. Random Forest was found to be more robust and easier to use. It was also found to perform better with high-dimensional datasets (28).

In recent years, many studies have been conducted using ML in the field of breast cancer. There were a few studies that focused on IDC and ILC differentiation using ML methods. In a study conducted in 2022 by Gunawan et al., a Support Vector Machine was used on 156 IDC and 8 ILC mammographic data to differentiate IDC and ILC (29). They selected 9 mammographic physical parameters of the image: entropy, contrast, MomentAng, MomentDiff, mean, deviation, EntropyHDiff, MomentHDiff, and MeanHdiff. Their model scored an accuracy of 76.56%. Maiti et al. did a study in 2024 in which he explored the texture features extracted from DCE-MRI images to differentiate IDC from ILC (30). These texture features included shape feature, gray level dependence matrix (GLDM), gray level co-occurrence matrix (GLCM), First order, gray level run length matrix (GLRLM), gray level size zone matrix (GLSZM), and neighboring gray tone difference matrix (NGTDM). The study was done on a dataset that included 58 patients (30 with IDC and 28 with ILC). They acquired 0.998, 97.21%, and 96.2% for Area under Curve(AUC), sensitivity, and specificity respectively. Faraz et al., extracted a total of 342 radiomic features from MRI images(DCE-MRI, subtraction and T2 weighted images) and selected 32 relevant features to differentiate IDC from ILC (31). They achieved an AUC of 0.73 when using SVM to classify a dataset of MR images from 323 patients.

Previous studies primarily focused on demographic data, laboratory-based data, and radiomic features of the tumor, with limited research on the morphological features of the breast (29–31). These studies were focused on the radiomic features of the tumor and mammographic physical parameters of the image. This has been the norm for the last few years. Prior to this, there were no studies in this field using only morphological features of the contralateral breast. Dimensions of the ipsilateral breasts, like total volume and fibroglandular volume, would vary because of tumors. Using threshold function on breast MRI images to delineate fibroglandular tissues would be influenced by the presence of tumors. Hence, contralateral breasts were chosen for this study. Bilateral breasts are considered physiologically almost identical, although slight asymmetries may be observable in most women. These asymmetries can manifest in size, shape, or volume and are typically within a normal range. Coltman et al. stated in their study that there was no significant difference in the mean breast volume between the women’s left and right breasts (32).

The aim of this study was to leverage ML techniques to develop a predictive model capable of accurately distinguishing between IDC and ILC, based on morphological features of the contralateral breast.

## 2 Methods

This retrospective, quantitative study included 143 female patients selected from a database of 922. The patients who were 40 years or older, had undergone a breast MRI examination, and were diagnosed with IBC. The cohort consisted of 117 patients with IDC and 26 with ILC, classified based on histopathological reports available publicly in the “DUKE Breast-Cancer-MRI” database from TCIA website. The patients who had undergone surgical intervention in the contralateral breast (CLB), those with bilateral breast cancer, MRI image artefacts, significant breast density asymmetry, known benign mass lesions in the CLB, or those lacking axial T1-weighted pre-contrast MRI images were excluded. The flowchart of the patient selection protocol is illustrated as **Supplementary Figure S1** in supplementary materials.

The Ethics Review Committee of the Faculty of Allied Health Sciences, University of Peradeniya, Sri Lanka, approved the study protocol. All procedures were performed per relevant guidelines and regulations.

**Figure 1:**
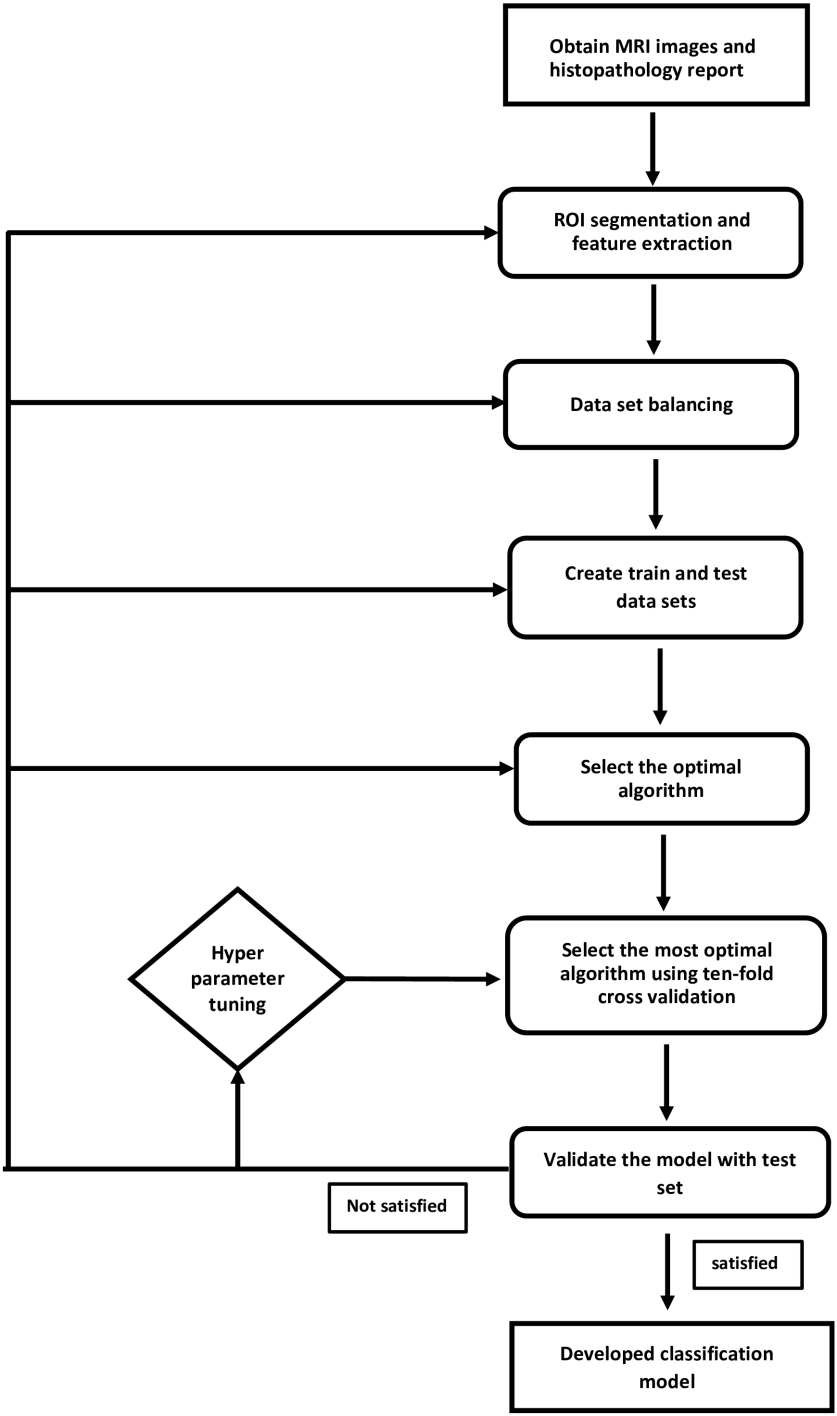
Supervised learning method to classify IDC and ILC was applied. This flowchart illustrates the steps followed to establish a prediction model.

Breast MRI images were sourced from the openly accessible “Duke Breast-Cancer-MRI” collection on the Cancer Imaging Archive website (33). This study adhered to The Cancer Imaging Archive’s data usage policy and restrictions. The DUKE database was collected at Duke Hospital, USA, between 2000 and 2014. Images were acquired using 1.5T or 3T scanner from General Electric and Siemens. Digital Imaging and Communications in Medicine (DICOM) images of the axial T1-weighted pre-contrast sequence were included in the study. The workflow of the supervised learning method used in the development of the IDC and ILC classification model is illustrated in **Figure 1**.

### 2.1 Image processing and region of interest selection

**Figure 2:**
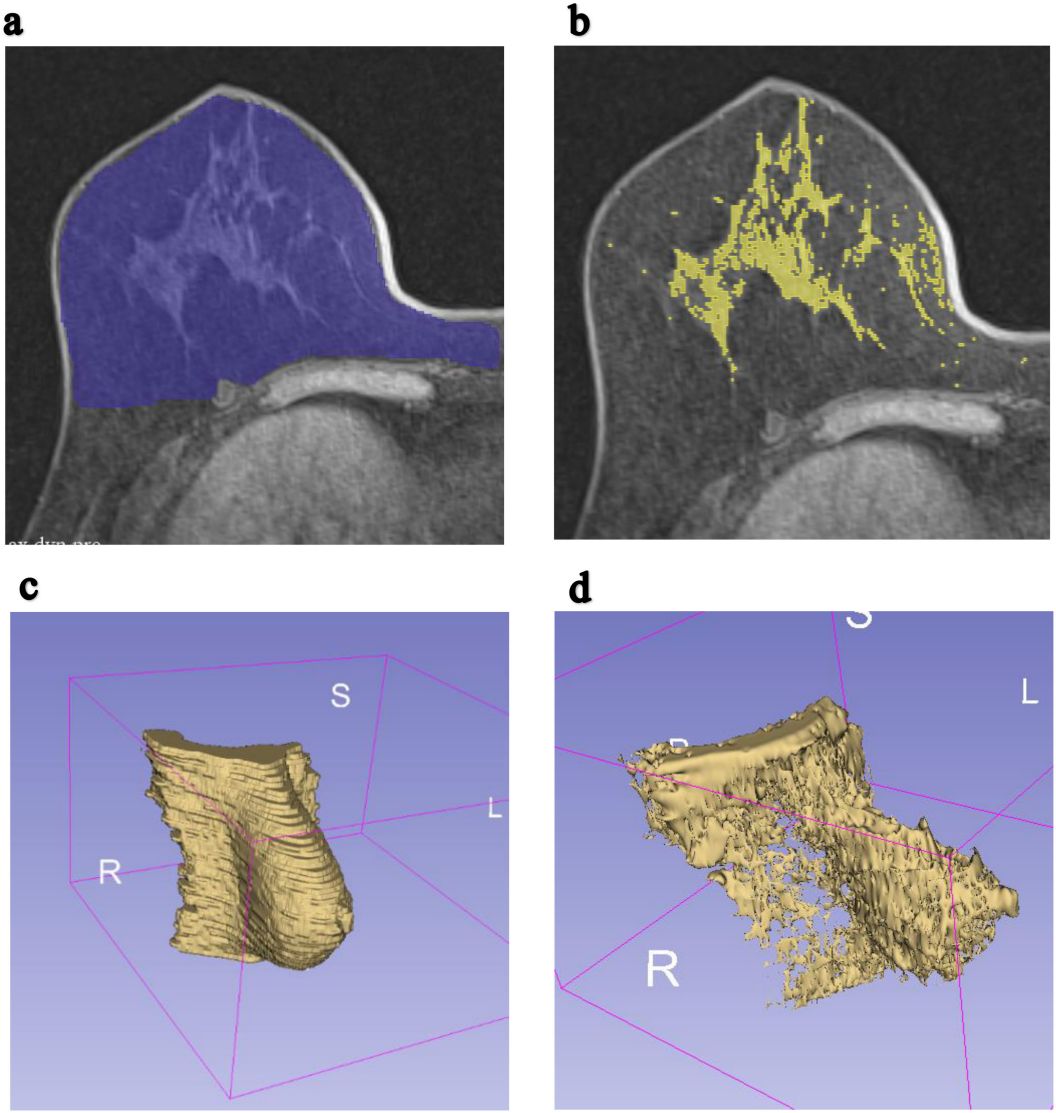
Example of MRI T1-weighted breast axial images were segmented to compute total breast volume and fibroglandular volume. **(a)** ROI for a single slice of the right breast in the axial plane. **(b)** ROI for a single slice of the fibroglandular tissue of the right breast in the axial plane. **(c)** The constructed 3D image of total volume of the contralateral breast. **(d)** The constructed 3D image of fibroglandular volume of the contralateral breast.

3D Slicer software (version 5.2.2) was used as the image processing tool. The investigator manually selected the ROI within the total breast area of the CLB as illustrated in **Figure 2**. The anterior border was defined by the skin margin, and the posterior border was delineated at the level of the sternum. Pectoralis muscles, identified by their grey color in MRI images, were excluded from the ROI selection process, with validation provided by a board-certified radiologist. The ROI for the fibroglandular area of the CLB was marked in each slice using the thresholding function of the 3D Slicer application and by manually delineating the fibroglandular area. A comprehensive 3D image of the CLB was generated by combining all the slices of the MRI image.

### 2.2 Quantification of morphological features

The 3D Slicer application’s quantification feature was used to determine parameters for each patient’s CLB. Specifically, the total volume of the CLB, fibroglandular tissue volume of the CLB, surface area of the CLB, and surface area of the fibroglandular tissue of the CLB were computed. MRI breast density, a crucial metric in breast health assessment, was calculated as the ratio of fibroglandular tissue volume to the overall breast volume. In addition, the ratio of fibroglandular tissue volume of the CLB, to the surface area of the fibroglandular tissue of the CLB, and the ratio of total volume of the CLB, to the surface area of the CLB were calculated.

### 2.3 Machine learning model development

After feature computation, supervised learning methods were applied to build a model for predicting types of IBC (IDC or ILC). The model was developed using Python 3.12 software. Given the imbalance between IDC and ILC cases, the Synthetic Minority Oversampling Technique (SMOTE) was applied to balance the dataset. The resampled dataset was stratified and split into training and testing subsets. Different proportions of training and testing data set combined with different feature selections were used to find the optimal splitting ratio(as shown in **Table 1**). A training and testing data set with the proportion of 80:20 was chosen, with all 7 features selected to build the model.

**Table 1:** Results from tenfold cross validation and test set validation based on various combination of train and test data splits and feature selection.

Testing set (%)	Features	RFC ten-fold cross validation score	Accuracy
15	4	77.13	72.22
	5	78.26	58.33
	6	81.78	66.66
	7	81.76	69.44
20	4	74.325	68.08
	5	75.46	70.21
	6	78.07	74.46
	7	79.12	70.21
25	4	72.38	69.49
	5	74.24	69.49
	6	75.94	72.81
	7	75.42	76.27
30	4	71.98	69.01
	5	71.36	74.64
	6	73.78	69.01
	7	70.73	69.01
35	4	69.16	63.41
	5	71.11	75.6
	6	71.83	69.51
	7	69.16	75.6
40	4	69.28	74.46
	5	71.42	76.59
	6	69.28	79.78
	7	70.71	79.78

### 2.4 Feature selection and model training

The ANOVA F-test was used to select a subset of normalized features most pertinent to distinguishing between IDC and ILC. All 7 features were selected based on a tenfold cross-validation method which was employed to evaluate various algorithms, including Support Vector Classifier (SVC), Random Forest (RF), Gaussian Naive Bayes (GaussianNB), K-Nearest Neighbors (KNN), Decision Tree Classifier (DT), Logistic Regression (LR), and Linear Discriminant Analysis (LDA). The RF classifier, which provided the best performance, was used to develop the final model. The selected RF classifier is then validated on the testing set.

### 2.5 Model optimization and evaluation

GridSearchCV is a sklearn technique which uses grid search algorithm to find the optimal hyperparameters for the model (34). These hyperparameters include min_samples_split, n_estimators, max_depth, max_features, bootstrap, and min_samples_leaf. Each hyperparameter was tested within predefined ranges; min_samples_split: from 2 to 5, n_estimators: from 100 to 200 (with the steps of 10), max_depth: 10 to 100(with the steps of 10),max_features: 3, 5, 10, boot_strap: True, False, min_samples_leaf: 1, 2, 4. Precision and recall scores of the model were acquired.

The model’s performance was evaluated using several metrics, including accuracy, precision, recall, and F1 score as stated here by the Equations 1–4.


(1) $$Accuracy=\frac{\left(TP+TN\right)}{\left(TP+TN+FP+FN\right)}$$

Where TP,TN,FP and FN specify true positive, true negative, false positive and false negative, respectively. Here, Accuracy represents the proportion of correct predictions out of the total number of predictions made by the ML model.


(2) $$Precision=\frac{TP}{\left(TP+FP\right)}$$

Where TP and FP represent true positive and false positive. Precision represents the proportion of the correctly made p positive predictions made by the ML model.


(3) $$Recall=\frac{TP}{\left(TP+FN\right)}$$

Where TP and FN indicate true positive and false negative. Recall indicate the correctly predicted positive outcomes out of all the positive individuals.


(4) $$F1\;=\;2.\left[\frac{\left(Precision.\;Recall\right)}{\left(Precision+Recall\right)}\right]$$

F1 score indicates the balance of precision and recall.

The precision-recall curve was used to illustrate the trade-off between sensitivity and specificity. The final decision threshold was set at 0.5 to maximize the model’s performance. The area under the ROC curve (AUC-ROC) was also evaluated to further assess the model’s efficacy.

## 3 Results

### 3.1 Feature selection and ANOVA F-Test Scores

The ANOVA F-test was used to identify the most significant features for distinguishing between IDC and ILC. As shown in **Table 2**, the feature “Total volume of the contralateral breast” reported the highest ANOVA F-test score (mean value = 8.343721), indicating a strong correlation with the IDC and ILC differentiation. In contrast, “Fibroglandular volume of the contralateral breast” (mean value = 0.001393) reported the lowest score, suggesting minimal correlation with the cancer types. Another notable feature is “Surface Area of the contralateral breast” with a score of 6.921108.

**Table 2:** ANOVA F-test scores of 7 morphological features used in the study for feature selection.

Feature	ANOVA F-test score
Total volume of the contralateral breast	8.343721
Fibroglandular tissue volume of the contralateral breast	0.001393
Breast density	6.382038
Surface area of the contralateral breast	6.921108
Surface area of fibroglandular tissue of the contralateral breast	0.817569
Fibroglandular volume of the contralateral breast/Surface area of fibroglandular tissue	1.735226
Total volume of the contralateral breast/Surface area of the contralateral breast	5.226712

### 3.2 Algorithm performance

Several ML algorithms were evaluated to determine the most effective model for predicting IDC and ILC. **Table 3** summarizes the accuracy and standard deviation of each algorithm based on ten-fold cross-validation.

**Table 3:** Performance summary of different algorithms under cross validation (mean accuracy and standard deviation).

Algorithm	Mean Accuracy (%)	Standard Deviation
Logistic Regression	56.6	0.128070
Linear Discriminant Analysis	57.16	0.121735
k-Nearest Neighbors Classifier	65.17	0.112232
Decision Tree Classifier	71.19	0.116184
Gaussian Bayes	63.65	0.131818
Support Vector Classifier	71.05	0.143569
Random Forest Classifier	79.13	0.096389

The Random Forest Classifier demonstrated the highest mean accuracy of 79.13%, outperforming other algorithms such as Decision Tree Classifier(71.19%), Support Vector Classifier (71.05%), and Gaussian Bayes (63.65%).This high performance, combined with a relatively low standard deviation, indicated the Random Forest Classifier’s robustness and reliability in distinguishing between IDC and ILC.

### 3.3 Model evaluation and performance metrics

The Random Forest Classifier, identified as the best-performing model, was further evaluated using the test set. The evaluation metrics included precision, recall, and F1-score for both IDC and ILC. **Table 4** presents the detailed performance metrics.

**Table 4:** Performance of the random forest classifier on the test set.

Cancer Type	Precision (%)	Recall (%)	F1-Score (%)	Support
Ductal	67	78	72	23
Lobular	75	62	68	24
Accuracy			70	47
Macro Average	71	70	70	47
Weighted Average	71	70	70	47

The Random Forest model achieved an overall accuracy of 70% with IDC and ILC classifications. ILC exhibited a higher precision score (75%) while IDC exhibited a higher Recall score (78%). The F1-score, which balances both precision and recall, is higher for IDC (72%) compared to ILC (68%).

### 3.4 Hyperparameter tuning and decision threshold adjustment

To further enhance the model’s performance, hyperparameter tuning and decision threshold adjustments were conducted. Precision and Recall scores were maximized by running GridSearchCV on the model to find the optimal hyperparameters within a pre-defined range. **Table 5** describes the details of optimized hyperparameters for maximizing precision and recall, respectively.

**Table 5:** Optimized hyperparameters for maximum precision and maximum recall.

Hyperparameter	Maximized precision	Maximized recall
Max Depth	20	30
Max Features Min samples leaf	3 1	7 1
Min Samples Split	2	2
Number of Estimators	133	177

After tuning, the Random Forest model with maximum precision correctly predicted 19 cases of IDC and 17 cases of ILC, with 4 false negatives and 7 false positives. The performance metrics for this optimized model are shown in **Table 6**.

**Table 6:** A summary of performance metrics for the model optimized for precision.

Cancer Type	Precision (%)	Recall (%)	F1-Score (%)	Support
Ductal	73	83	78	23
Lobular	81	71	76	24
Accuracy			77	47
Macro Average	77	77	77	47
Weighted Average	77	77	77	47

Precision, Recall, F1-score and support score were obtained.

For maximum recall, the model accurately predicted 19 cases of IDC and 18 cases of ILC, with 4 false negatives and 6 false positives. The performance metrics are detailed in **Table 7**.

**Table 7:** A summary of performance metrics for the model optimized for recall.

Cancer Type	Precision (%)	Recall (%)	F1-Score (%)	Support
Ductal	76	83	79	23
Lobular	82	75	78	24
Accuracy			79	47
Macro Average	79	79	79	47
Weighted Average	79	79	79	47

Precision, Recall, F1-score and support score were obtained.

**Figure 3:**
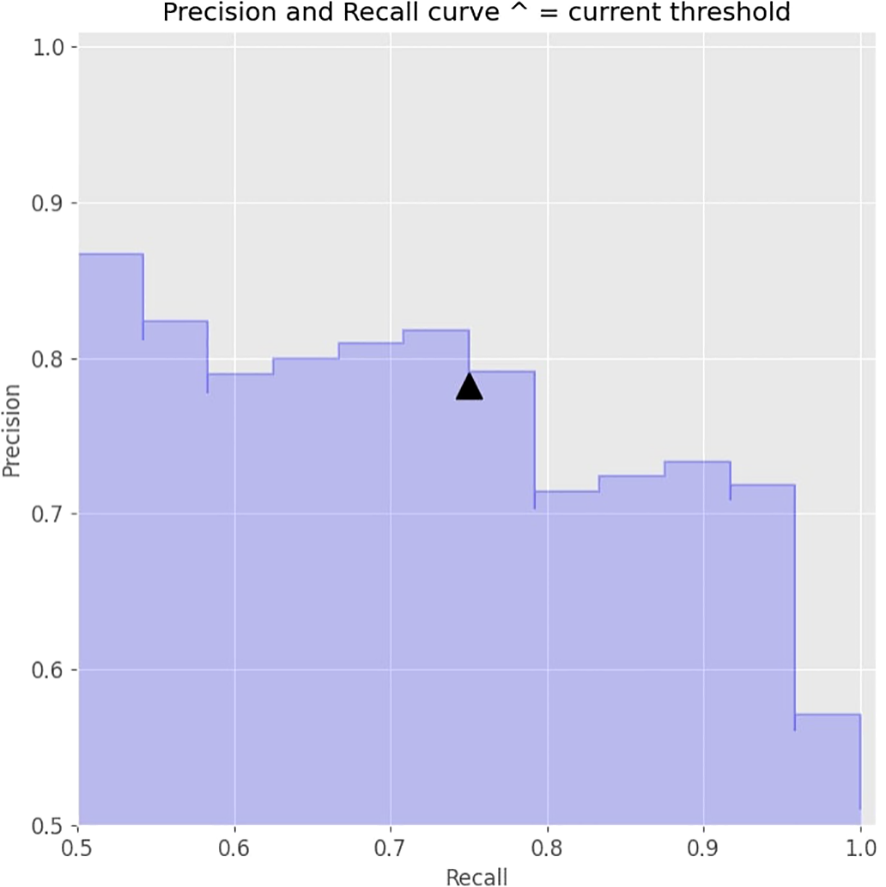
Precision- recall curve of the machine learning model with threshold level.

Further adjustments were made to the decision threshold based on the precision-recall curve (illustrated in **Figure 3**), which optimized the balance between precision and recall. The decision threshold was adjusted to 0.5 to achieve optimal values. The final model achieved an accuracy of 79%.

**Figure 4:**
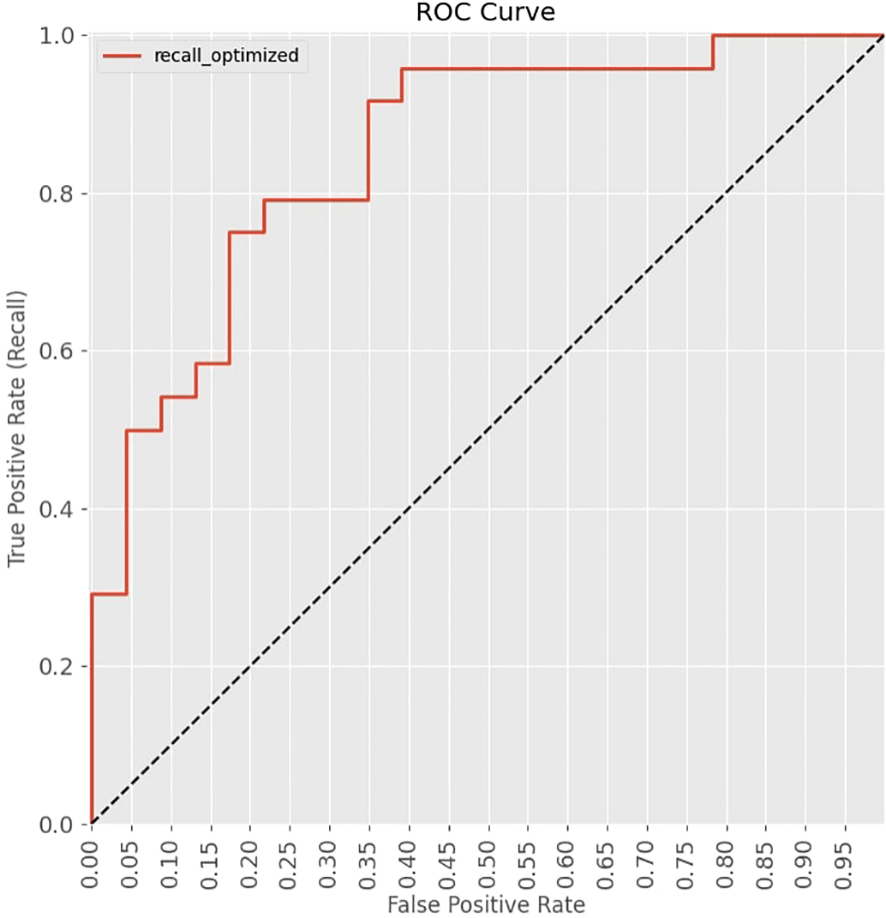
Receiver Operating Characteristic (ROC) Curve of the machine learning model.

The ROC curve, shown in **Figure 4**, plots the true positive rate against the false positive rate across different decision thresholds, providing insight into the trade-offs between precision and recall. An AUC value of 0.851 was obtained which indicates the model’s ability to distinguish positive and negative outcomes.

**Figure 5:**
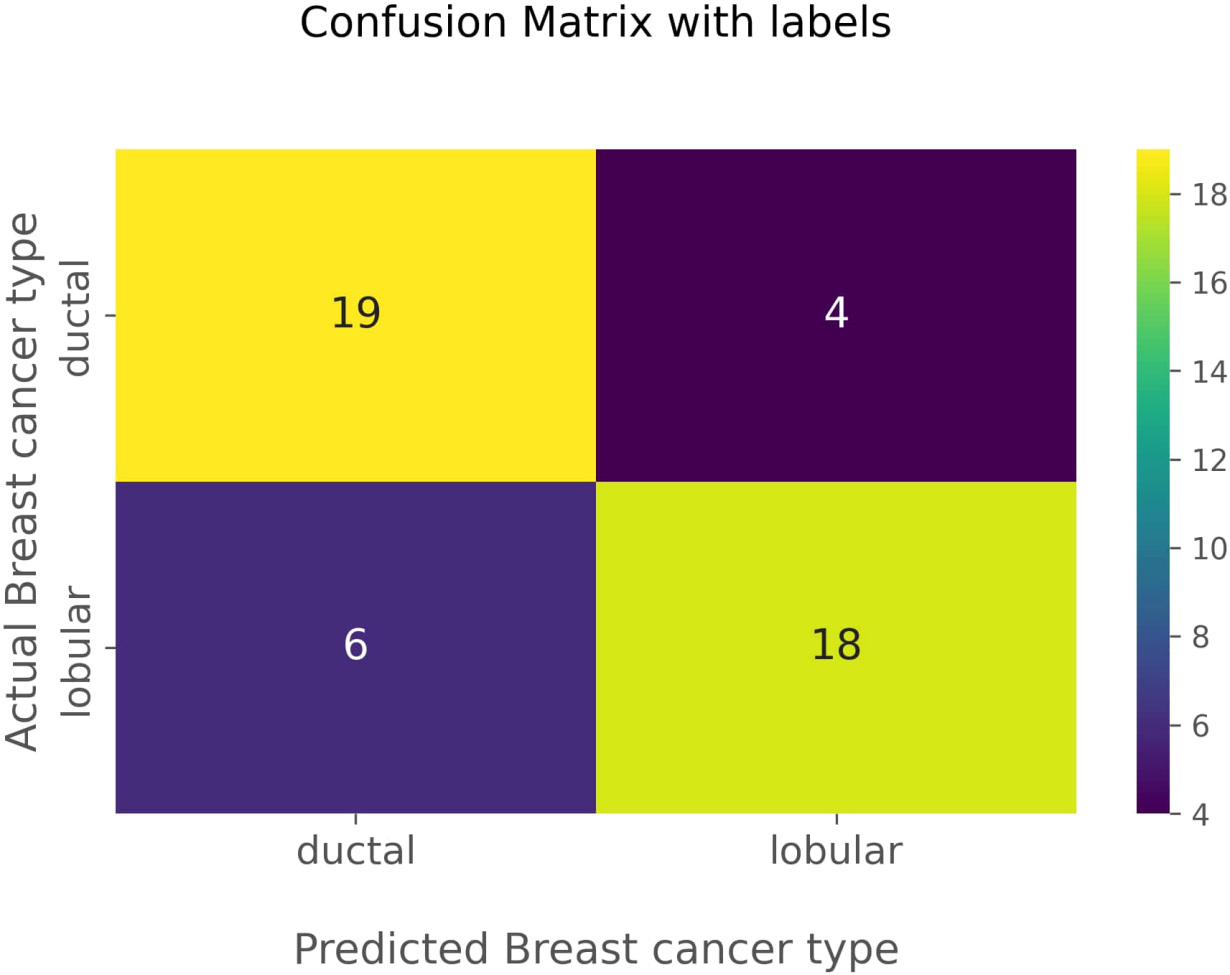
Confusion matrix of machine learning model with predicted and actual cancer types.

The confusion matrix in **Figure 5** visually represents the performance of the optimized classification model. The matrix highlights the 19 true negatives, 18 true positives, 6 false positives, and 4 false negatives. In this aspect, true negatives and true positives are identified as correctly predicted IDCs and correctly predicted ILCs, respectively. False positives are identified as actual ILCs, falsely predicted to be IDCs, and false negatives are identified as actual IDCs, falsely predicted to be ILCs. The confusion matrix corroborates the model’s high accuracy and balanced performance in distinguishing between IDC and ILC.

## 4 Discussion

Past studies incorporating the RF Classifier in building ML models for breast cancer prediction reported various accuracies and sensitivities (32, 35). However, they focused entirely on the affected breast, while the focus was on the contralateral breast in this study. Among the morphological features extracted from the contralateral breast, total volume of the breast, surface area of the breast, and breast density had higher ANOVA F-test scores, indicating higher predictive power when differentiating IDC and ILC. A study by Egan et al. observed a significant positive association between breast size and breast cancer (36). Wanders et al., in their study, found a correlation between breast density, and both interval and screening breast cancers (37). A study by Nara et al. found a significant correlation between fibroglandular volume and breast cancer (38). Past studies related to classifying IDC and ILC primarily focused on the tumor’s radiomic features, and they did not consider the morphological features of the breast. Gunawan et al. acquired an accuracy of 76% based on mammographic physical parameters (29). A study by Faraz et al. focused on radiomic features to achieve an AUC of 0.73 (31). We have selected morphological features of the contralateral breast in contrast to their studies and achieved a modest accuracy of 79% and an AUC of 0.851. Compared to their models, our machine learning model performed slightly better in differentiating IDC and ILC. A study by Maiti et al. achieved an AUC of 0.998 for building a model based on the radiomic features of the tumor (30). Compared to other studies, they achieved an impressive AUC but didn’t use machine learning or deep learning methods because of the smaller sample size. It can be reasoned that using ML model would not be ideal for smaller datasets like our study. In 2023, Bouasria et al. explored the optimal sample size needed for a RF model (39). They mentioned that a 300–500 sample size was recommended for optimal performance, but the accuracy difference above 50 sample size was shown to be minimal, not accounting for deeper analysis. Hence, the ML model could be advantageous since it could handle high dimensional datasets and precision-recall tradeoffs. Contralateral breast cancer refers to development of cancer in the opposite breast after a patient has already been diagnosed with cancer in one breast. Giannakeas et al., in their study observed that 3.2% of patients were diagnosed with the CLB cancer, and the 25-year actuarial risk of contralateral invasive breast cancer was 9.9% (40). There are several risk factors for the CBC cancer- age, genetic mutations, tumor type and hormone receptors. Several studies have discussed correlation between histological type of primary breast cancer(IDC and ILC), and the development of contralateral breast cancer (41, 42). Langland et al. observed that there were no risks in developing contralateral breast cancer based on primary cancer histology(IDC and ILC) (42). However, a study by Park et al., based on a retrospective analysis of 1071 patients found that the risk of developing contralateral breast cancer was higher in patients with ILC(7.1%) compared to those with IDC(1.5%). This finding aligns with an earlier study by Arpino et al. in 2004 which observed that the incidence of CLB cancer was higher in ILC patients than in IDC patients (20.9% vs 11.9%) (11). Therefore, our study could be extended in the future to explore relationship between the morphological features of the CLB, histological types of primary cancer(IDC and ILC), and the risk of CLB cancer.

We had an unequal number of IDC and ILC patients, with 117 and 26, respectively. The SMOTE oversampling method was used to balance the data set. The sample size of ILC was equalized to that of IDC, since IDC had the largest sample size in the population. Without balancing the dataset, ANOVA feature selection scores of 0.440811, 0.000123, 1.012839, 0.280833, 0.009705 and 0.198752 were obtained for total volume of the contralateral breast, fibroglandular volume of the contralateral breast, breast density, surface area of the contralateral breast, surface area of fibroglandular tissue, fibroglandular volume of the contralateral breast/surface area of fibroglandular tissue and total volume of the contralateral breast/surface area of the contralateral breast, respectively. The scores were very low across all the features and showed no distinct variations compared to the ANOVA F-test scores we obtained (see **Table 2**) after using the SMOTE oversampling method. Using SMOTE positively increased the performance of the ML model. When splitting train and test data, we had to go through all the combinations, as pointed out in **Table 1**, to find the ideal combination to build a robust model. Even though split ratios of 65:35 and 60:40 gave higher accuracies, the accuracies decreased significantly after hyperparameter tuning. Therefore, an 80:20 ratio was selected to reduce the variance and overfitting of the data. Overfitting is when the model performs exceptionally well on the training data set and poorly on the testing data set. Overfitting happens when the model tries to memorize the data instead of generalizing it in the training phase. Optimal hyperparameters were selected for the maximized precision and recall scores. Given the need to address IDC and ILC, it is essential to balance false positives and false negatives to optimize the performance of the machine learning model. Hence, precision and recall were maximized, and the threshold was set to 0.5 to acquire balanced precision and recall.

Based on this study, IDC and ILC can be differentiated without invasive procedures, which would immensely benefit medicine. This could complement biopsy procedures moving forward. In situations where performing biopsies are considered challenging, this model could be of service in such circumstances. It should be evaluated on a large data set in the future to be considered reliable. Data acquisition can also be accomplished quickly since we only focus on the fat-suppressed T1 weighted imaging. This can be achieved within minutes, and patient comfort is not compromised. The acquisition takes approximately 1 minute for standard resolution and 6 minutes for higher resolution at 3T (43).

However, there are several limitations in this study. The difference in the dataset sizes of IDC and ILC could have affected the model’s performance. Gunawan et al. also faced the same struggle with an unbalanced dataset(156 IDC and 8 ILC), which resulted in almost identical accuracy to our study (29). Decreasing the number of IDC cases to balance the dataset would also affect the model, as the ability to learn complex patterns would be compromised. As stated by Bouasria et al., decreasing the overall sample size to around 50 would marginally decrease the model’s accuracy (39). Hence, we proceeded with 117 IDCs and 26 ILCs as our dataset. Even though data acquisition is quicker with fat-suppressed T1 weighted imaging, the time needed to extract the morphological features of the breast would still be longer when using 3D Slicer. Multiple recent studies have focused on building automatic segmentation models based on deep learning methods like deep convolutional neural networks (44–46). These automatic segmentation methods could be incorporated into our study to speed up the segmentation process further. Further advancements should be made to expedite the acquisition of morphological features of the breast. Another limitation of this machine learning model is the lack of an external data set comprising IDC and ILC patients for validation. External and multi-center validation is needed to gauge the accuracy and reliability of the model across various clinical settings. It would also prevent the overfitting of the model. Therefore, future studies should focus on incorporating external validation into this model.

## 5 Conclusion

This study developed and optimized a predictive model using morphological features of the contralateral breast and ML methods to differentiate IDC and ILC. The RF Classifier was identified as the most effective algorithm, achieving a high level of accuracy through extensive feature selection and hyperparameter tuning.

The significant predictive power of features such as the total volume of the CLB, surface area of the CLB and breast density underscores the importance of comprehensive feature selection in developing robust ML models. Classification of IDC and ILC through non-invasive method like this could be further developed in the future to complement biopsies in challenging situations.

Future directions should include expanding the dataset size and incorporating additional features to enhance model accuracy. By improving diagnostic accuracy, the developed model represents a valuable advancement in breast cancer management, ultimately contributing to better patient outcomes and more efficient healthcare delivery.

## Data Availability

The raw data supporting the conclusions of this article will be made available by the authors, without undue reservation.

## Supplementary material

Supplementary Figure 1 This flow chart illustrates the patient selection protocol from “Duke Breast-Images-MRI” data base in TCIA website.
