# Supplementary material for: Machine learning-based classification model to differentiate subtypes of invasive breast cancer using MRI
Source: Front Oncol. 2025 Jun 3;15:1588787. doi: 10.3389/fonc.2025.1588787 (PMC12170307; doi:10.3389/fonc.2025.1588787)
Supplement: Supplementary Figure 1 — This flow chart illustrates the patient selection protocol from “Duke Breast-Images-MRI” data base in TCIA website. [file Table1.docx]

**Supplementary material**

**
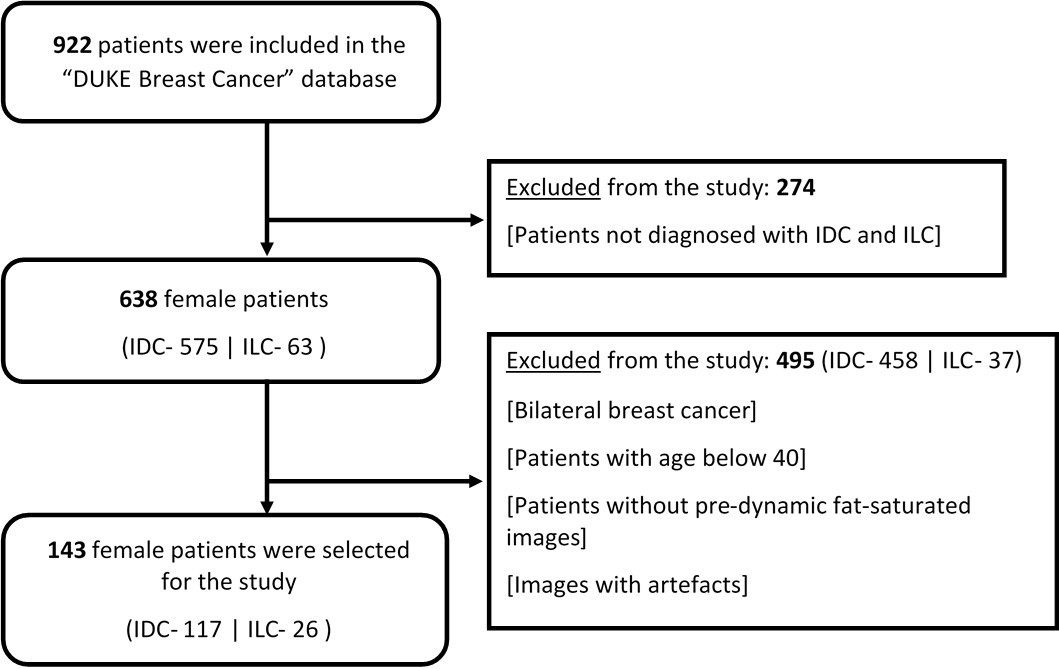
**

Figure 1S: This flow chart illustrates the patient selection protocol from "Duke Breast-Images-MRI" data base in TCIA website
